# Advancing emigrants' rights in India: strategies of civil society in spaces for engagement

**DOI:** 10.1080/14747731.2024.2384147

**Published:** 2024-07-31

**Authors:** Mira Burmeister-Rudolph

**Affiliations:** Department of Political Science, University of Amsterdam, Amsterdam, The Netherlands

**Keywords:** Civil activism, civil society, migrant workers' rights, transnational precarious labour

## Abstract

This article explores Indian civil society's efforts to promote the rights of migrant Indian labourers working abroad in low-wage employment as a response to weakly institutionalized rights frameworks in the global and India's national governance of labour migration. Existing scholarship has explored civil societies' advocacy in multi-national fora, at regional levels, and as forms of transnationally organized networks, but only marginally at the analytical level of migrant-origin states. The article examines their multi-level and multi-stakeholder strategies through the analytical lens of spaces for engagement. Drawing on original qualitative data, this article shows that civil society organizations play a leading role in (e)migration governance in India and are key to understanding the politics of migration of the world’s largest migrant-origin state. In particular, the local and subnational levels are important entry points to advance their agendas in the context of migrant-origin states.

## Introduction

This article investigates how Indian civil society stakeholders utilize and create ‘spaces for engagement’ (Bisong, 2022, p. 2946) and their related strategies to advance the human, labour, and citizen rights of Indians who migrate into low-wage labour employment abroad. More than half of the total of all Indian emigrants migrate to the Gulf Cooperation Countries (GCC) and a majority of them work in low-wage employment (Khadria, 2006) on temporary work contracts under the kafala system, a sponsorship system which is regarded as prompting exploitative practices (Thiollet, 2019). However, the state’s transnational protection policies are weakly institutionalized: they are programmatic and not enshrined in law; the labour agreements with destination countries in the GCC region do not cover dimensions of protection (Wickramasekara, 2012), and India has not signed the UN Convention on Migrant Workers (United Nations, 2023).

A global body of literature has demonstrated that it has been civil society organizations (CSOs)^1^ who have played a pivotal role in setting origin- and destination-state governments’ agendas on migrant issues and championing a rights-based framework (e.g. Likić-Brborić, 2018; Piper, 2015; Rother, 2022) – policies and measures aimed at providing stronger legal protections, ensuring equitable access to decent work opportunities, and establishing robust social safety nets. Their efforts have been a response to the shortcomings in various critical aspects of the governance of labour migration despite a rise in international cooperation on migration, leading to significant deficits in terms of decent work, labour rights, employment prospects, and social protection. Instead of a rights-based framework, migrant origin and destination countries, and International Organizations (IOs) have championed managerial/developmental approaches, which aim at steering migration flows to optimize their utility in terms of development through remittances for origin countries and the supply of ‘regular’ migrants to fill labour market shortages in destination countries (Pécoud, 2021).

Drawing on data from semi-structured interviews with civil society stakeholders in India conducted between early 2020 and 2023 and observation of their activities through in-person participation and on social media, this paper examines how civil society stakeholders aim to achieve social justice for migrants through influencing policymaking and practices of service provision. In line with scholarship that has attributed civil society as a major contributing factor in the setting of standards for labour emigrants (see Rother, 2022), I argue that they play an essential role in (e)migration governance in India and are therefore central to understanding the politics of migration of the world’s largest migrant-origin state. CSO’s role might not come as a surprise given its general centrality in both lobbying for and providing services to marginalized groups within India (see Chandhoke, 2012). This research complicates the notion of CSOs as solely being trailblazers in promoting migrant rights, revealing their role to be more ambiguous in relation to stakeholders, such as IOs, and emigrants.

This article contributes to the growing literature on ‘bottom-up’ approaches (Piper, 2015) to studying (labour) migration governance by centring on actor-oriented perspectives. The analysis focuses on civil society’s interactions with the local, regional, and national levels of the Indian state while acknowledging that labour migration is intrinsically transnationally organized and thus policymaking, activism, and advocacy work is likewise taking place across multiple scales. It thereby focuses on the analysis of strategies, but not on their outcomes or results. By describing CSOs’ engagement with the multiple levels of the migrant-origin context, this research adds to existing scholarship which predominantly has explored civil societies’ advocacy in multi-national fora, e.g. at the GMFD and GCM (Likić-Brborić, 2018), regional levels, such as ASEAN (Rother, 2022) or ECOWAS (Bisong, 2022) and as forms of transnationally organized networks (Piper & Rother, 2020, 2022), but only marginally at the analytical level of migrant origin states. It demonstrates that particularly the local and subnational levels are important entry points for CSOs to advance their agendas in the context of emigrant origin states – in line with what we know about immigration contexts (see, e.g. Ambrosini, 2013).

The first part of this article discusses the conceptual underpinning of the analysis – spaces for engagement, strategies of advocacy and activism, and CSOs as mediators between various institutional levels, citizens and the state. Next, I outline my research design, including the data collection process and analysis. In the subsequent empirical sections, I first provide an overview of the Indian context of migration for low-wage employment and the role of CSOs therewithin, before examining their multi-level and multi-stakeholder strategies in spaces for engagement.

## Bridging gaps: CSOs as mediators of migrant rights in multi-scalar political spaces

To understand how Indian civil society actors aim to achieve their objectives of bettering migrant rights, this article brings together the concepts of spaces for engagement (Ålund & Schierup, 2018; Bisong, 2022; Miraftab, 2004), mediated citizenship (Piper & von Lieres, 2015), and scalar politics (MacKinnon, 2011) which shed light on CSOs’ strategies in engaging with policy processes, and their mediating role between different institutional levels, citizens, and the state. Scholarship analyzing the engagement of CSOs in global and regional migration governance has highlighted the centrality of ‘spaces for engagement’ (Bisong, 2022, p. 2946) to understand their strategies and roles (Ålund & Schierup, 2018; Bisong, 2022; Piper & Rother, 2022; Rother, 2009). In this article, I build in particular on Miraftab’s (2004), Ålund and Schierup’s (2018), and Bisong’s (2022) concept of invited, invented, and instrumentalized spaces to analyze the interactions of CSOs within India’s multi-scale political system.

‘Invited spaces’, are platforms and opportunities for CSOs to actively interact and collaborate with the state at local, national, global, or transnational institutional levels, which aim at creating higher levels of citizen participation (Miraftab, 2004). These invited spaces often open up during the validation process before the adoption of policies when CSOs can voice their assessment or as a more institutionalized form of cooperation, through memoranda of understanding (MOUs) (Bisong, 2022). Scholars point to the risk of co-optation within these spaces, where civil society replaces the role of the state, as the state has withdrawn from public service delivery (Ålund & Schierup, 2018). Bisong (2022) calls these cases ‘instrumentalized spaces’, where CSOs may function as instruments of the state, IOs, or donor agencies to propagate messaging that aligns with the latter’s respective goals, e.g. through implementing particular policies. CSOs engage in such cooperations because of their access to funding, ideology, or position (Bisong, 2022), or on a grassroots level as a coping mechanism to deal with social and political inequalities (Miraftab, 2004). Consequently, co-optation for CSOs significantly influences their activities and objectives and ability to criticize policies or practices as well as acts to legitimize government and organization’s agendas (Ålund & Schierup, 2018; Bisong, 2022). However, ‘instrumentalized spaces’ also offer CSOs platforms to negotiate, criticize and potentially transform migration governance objectives, as Marino et al. (2023) in their study of Gambian-owned CSO's in the implementation of externalization policies show. Lastly, ‘invented spaces’ are alternative, independent spaces that CSOs create when they are excluded from participating in official platforms or to raise topics that are left out from these platforms’ agendas, thus formulating alternative policies. They attempt to challenge the existing status quo, aspiring to bring about broader societal change and resistance against prevailing power dynamics (Miraftab, 2004). CSOs use these spaces to build alliances with CSOs with similar views and aims (Bisong, 2022). Emerging from invented spaces, collaboration with state actors is possible in the form of complementary activities, where CSOs act, e.g., as technical partners (ibid.). Invented spaces on a regional or global level also allow CSOs to raise issues that are ignored on the national level and thereby create pressure for change from outside by other states, IOs, or CSOs (Bisong, 2022; Piper & Rother, 2022), what Keck and Sikkink (1998, 13) call the ‘boomerang’ pattern.

The literature on non-state actors’ strategies makes commonly a distinction between outside and inside strategies. Inside strategies involve direct interaction with decision-makers, such as meeting with them and offering policy expertise (Dellmuth & Tallberg, 2017), often described as advocacy work (see Fuller & McCauley, 2016). Outside strategies – associated with activism (see Fuller & McCauley, 2016) – try to influence decision-makers indirectly through public-opinion mobilization: e.g. mobilizing public opinion through news media, social media, and public events such as campaigns and protests (ibid.). With view to the previously described conceptualization of political spaces in the realm of civic activism, CSOs often predominantly use outside strategies in invented spaces or informal arenas (see Miraftab, 2004), and inside strategies for invited and instrumentalized spaces or formal arenas (ibid.).

Scholarship on social movements points to how the degree of permeability of political opportunity structures shapes social movement strategies, with closed or restrictive spaces of political participation leading to higher levels of outside engagement and more open spaces leading to strategies of inside engagement (see Kriesi, 2004). CSOs in India operate in a plurality of spaces, including local, subnational, national, transnational, and international. Political opportunity structures may differ on these scales, as may the types of spaces for engagement and strategies employed by CSOs, as well as their active efforts to seek out specific scales, such as local ones, as these offer open structures. As a result, CSOs strategically change register to engage in scalar politics (MacKinnon, 2011), in which stakeholders strategically deploy scale as part of their political activity.

CSOs utilize diverse tools and mobilization strategies throughout their endeavours (Miraftab, 2004). According to this understanding, this article does not differentiate between activist and advocacy organizations because the organizations under study cannot be neatly classified as either. In practice, this means these organizations cover activities focusing on the service delivery regarding immediate needs and finance stability in the sense of economic livelihood organizations and giving pre-departure training and information about the migration process; taking on the representation of a group to political authorities, such as through lobbying efforts, but also use activist strategies, e.g. developing the political agency of a group.

A large body of literature on transnational advocacy networks has highlighted the role of domestic CSOs as interlocuters or mediators of policy principles and global norms from the international domain to domestic contexts (Henry & McIntosh Sundstrom, 2021). Given that CSOs can be active both on domestic and international scales, I extend the definition of CSOs as mediators by building on Piper and von Lieres (2015) framework of ‘mediated citizenship’ to capture the dimension of CSOs’ interaction with migrants, their families, and the state. Mediation involves a third party’s effort to bridge or exploit the representational gap between disadvantaged and marginalized groups and the state. Intermediaries have multiple objectives: representation of a group to convey its interests to political authorities; advocacy by empowering the group politically by helping them develop the capacity to express their voices effectively securing their role and protecting their interests in the process (Piper & von Lieres, 2015). Representation through CSOs is contested (Piper & von Lieres, 2015): it exists as an unofficial procedure lacking endorsement from legal structures, questioning CSOs’ legitimacy and accountability (Bisong, 2022). While aiding migrants in precarious situations to attain their rights, it perpetuates social hierarchies.

As I will demonstrate in this article, domestic contexts shape how spaces for engagement are configured and how CSOs pursue their objectives; in the case of India, particularly the absence of the state is decisive. An analysis of the context-specific development of invented, instrumentalized, and invited spaces shows that they overlap. For example, participating in invited formal spaces of representative democracy, such as elections, by setting up female candidates from often marginalized migrant families can be a form of protest challenging the common pattern of nominating individuals who showcase substantial wealth and are often male and upper caste (see Jensenius, 2016), and therefore a way to claim space to make voices heard. Before turning to the empirical section, next, I will lay out the methodological approaches underlying this article and then describe the setting that makes CSOs key actors in the demand for emigrants’ rights in India.

## Methods and material

**Figure 1.**
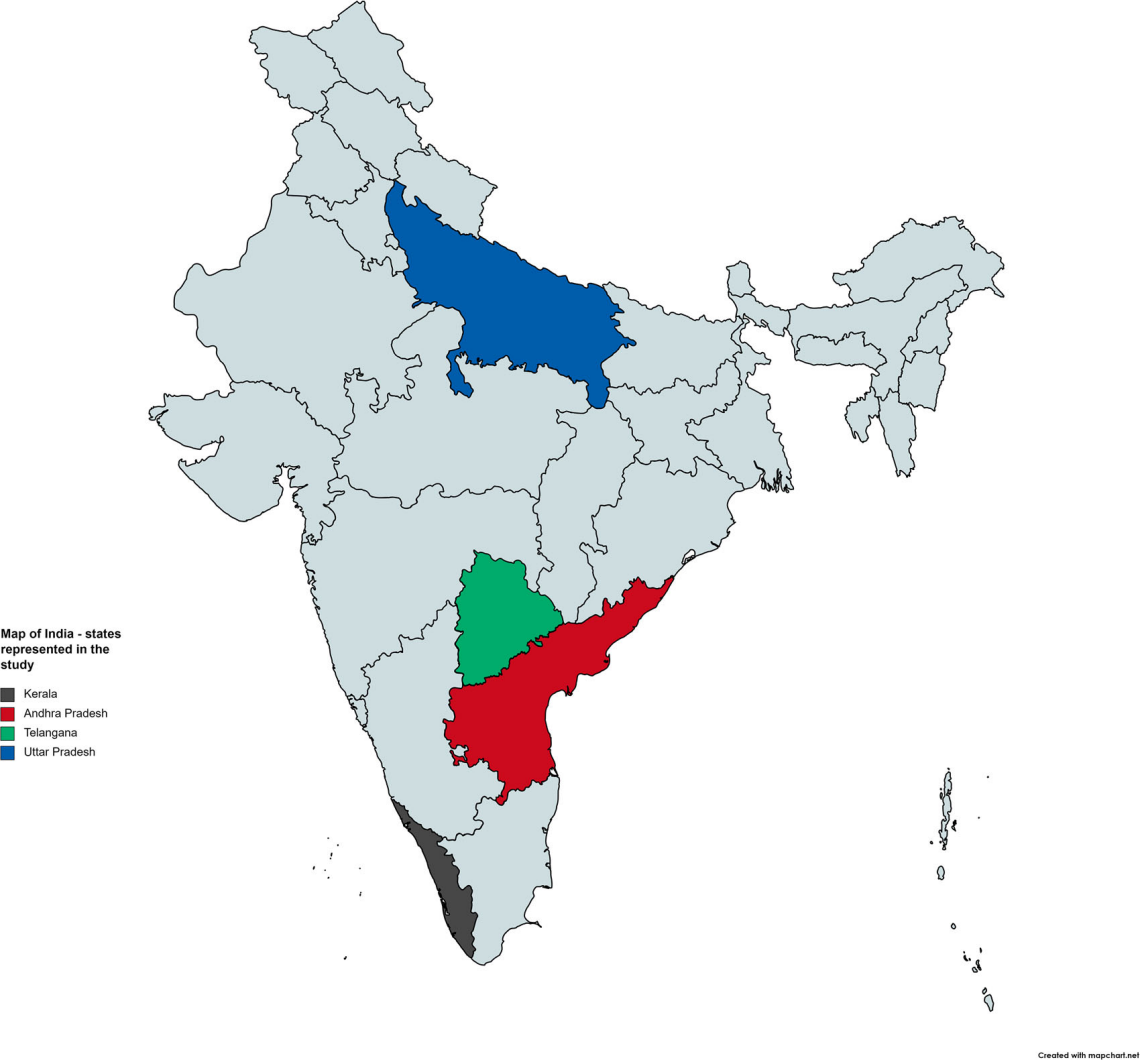
States represented in the study.

This paper analyses the spaces for engagement civil society organizations in India utilize and create and their related strategies of advocacy and activist engagements on behalf of emigrants migrating into low-wage employment, primarily to the GCC countries. The CSOs are located in India’s most prominent emigrant-origin states to the GCC countries: Kerala, Andhra Pradesh, Telangana, and Uttar Pradesh (see Figure 1). Geographically, post-independence emigration shifted from South Indian states to North Indian states. From the 1970s until 2009, the South Indian states of Kerala and Tamil Nadu were the largest major origin states for low-wage labour emigration (Kumar & Rajan, 2014). Since 2009, Uttar Pradesh has been the largest origin state of Indian low-skilled emigrants (Kumar & Rajan, 2014), while Telangana and Andhra Pradesh have become significant origin states too.

This article draws on multi-sited qualitative data collected between March 2020 and March 2023 in India, including interviews, both online and in-person; observations of meetings and interactions of CSOs with state officials, village-level political bodies, and migrants; and primary sources such as brochures, policy recommendation and petitions written by CSO stakeholders. Both in 2020 and 2023, I identified and reached CSOs through snowballing, using my network of respondents, colleagues, and acquaintances. The only available list on emigrants’ rights organizations is from Migrant Forum Asia’s website. The Indian Ministry of External Affairs (MEA), which provides lists of (selective) Indian migrant organizations in destination countries, does not offer a similar for domestic organizations. The limitation of this study is, therefore, that it might not cover the full breadth of organizations engaged with emigrants’ rights. Interestingly, however, respondents – whom I reached through different initial ‘gatekeepers’ and who were located in various Indian states as well as in countries outside of India – usually mentioned the same organizations, indicating a small network of organizations. In comparison, organizations working on internal migration in India officially figure much higher, with UNESCO (2013, p. 57) stating that more than 30 organizations are ‘involved in providing recommendations for mainstreaming concerns of migrant workers in policy’ and providing access to basic entitlements (ibid).

In pre-pandemic India, I held three in-person interviews with individuals who have been active in transnational migrant advocacy groups that support migrant workers and their families in the pre- and post-migration stages. In early 2023, I conducted three online interviews and one in-person interview with CSO representatives. In addition, I spent several days with a CSO in Uttar Pradesh, which allowed me to be part of their meetings and interactions with officials of the state’s Non-Resident Indian (NRI) department, panchayats (political unit at village-level) and Non-governmental Organizations (NGOs) active in rural areas as well as migrants and their families (see Table 1).

**Table 1.** Organizations in the study.

Location	Target audience beyond international migrants	Affiliation	Funding
Kerala	Migrants’ families, domestic workers	Catholic church	Catholic church
Kerala	Migrants’ families	MFA	Unknown
Hyderabad/Andhra Pradesh	Migrants’ families, domestic workers	Catholic church	Catholic church IOs (project based) Andhra Pradesh government (pre-departure training)
Telangana	Migrants’ families	Unknown	Donations, membership fees
Uttar Pradesh	Migrants’ families, internal migrants, migrants from Nepal (trafficking), children, women, homeless	Bharat Janata Party (ideational support)	IOs (project based), Donations
Uttar Pradesh	Internal migrants	Trade unions	Membership fees
Delhi	Workers (construction & wood)	Global union federation	Membership fees

To learn about the strategies of activism and advocacy, my interview guide questions and observations aimed to capture respondents’ interactions with other stakeholders, such as IOs, other CSOs, and Indian state institutions on multiple administrative scales, and the forms of these interactions, e.g. I asked about the organization’s objectives and how they aim to achieve the former. I used an abductive approach that focuses on the interactive and iterative process of data collection, data analysis, and theory-building. Whereas deductive approaches are theory-driven and inductive approaches develop from the empirical material, abductive analysis uses both approaches simultaneously (see Schwartz-Shea & Yanow, 2012). I initially coded using categories based on the interview questions and my observations and then built categories inductively from the data during the coding process and deductively from relevant literature.

I follow a positionality approach in migration research that goes beyond the ‘insider’–‘outsider’ dichotomy (Carling et al., 2014), reflecting on how settings and researcher-respondent dynamics affect social markers and perceptions of difference. My ascriptions include being female, young, White, middle-class, holding a European passport, and being affiliated with a prestigious European university. My European passport facilitated fewer hurdles of obtaining a visa and shorter visa waiting times compared to, for instance, an Indian PhD researcher (Sharma, 2023). In the research context, less obvious social markers, such as migration experience, language skills, and partnership/marriage status, which I could navigate more strategically because of their invisibility, also influenced interactions. Most of the interlocutors I would categorize as male, often also older than me. Compared to these interactions, engaging with women was easier. Some female respondents closer to my age made an effort to stay in touch, which I saw as a form of female bonding. The seniority, gender, and middle/middle-upper class status of the respondents shaped power dynamics in our interactions. Being female and young (often perceived as younger) made me seem less threatening and easier to trust (see also Glasius et al., 2018). The ascription of being foreign and White also affected interactions with interlocutors. To build familiarity and trust, I highlighted my knowledge of Indian politics and migration dynamics and mentioned my extended stays in different parts of India.

## CSOs in India and their role in the migration policy process

Piper and von Lieres’s (2015) framework points to why and how CSOs take on intermediaries’ roles in domestic contexts of migration governance. While the literature on CSO’s role in regional or global fora points to the demand for spaces of participation (Bisong, 2022), analyzing domestic levels makes it necessary to scrutinize the specific constellation of state-citizen relations and asks why migrants do not act on their own behalf. In most cases of South-South temporary contract migration, low-wage migrants do not only experience exploitative working and living conditions in the destination countries, but precarity already marks their lives before migration in terms of social-economic marginalization (Deshingkar, 2019): prospective low-wage migrants often work in the informal economy in India, inflected by caste, religion, and gender inequalities (Jayal, 2009). The central role of CSOs in India for rights advocacy and service delivery is not limited to the realm of migration governance but also holds for other policy fields. Intermediaries, including CSOs, are vital institutions bridging the gap between the state and rural communities in India, especially for socio-economically marginalized citizens who perceive the state as distant (Krishna, 2011), and have also transformed into an alternative entity to the state itself (Chandhoke, 2012).

The CSOs under study vary regarding their organizational affiliations. They are mostly grassroots organizations and cover NGOs, migrants and migrant returnees, faith-based organizations, but also include trade unions and union federations. According to their affiliation, the CSOs are funded e.g. by the Catholic church, union membership fees, donations, or IOs, such as United Nations (UN) Women, the International Labour Organization (ILO), and the International Organization for Migration (IOM), particularly for projects (see Table 1). Partially, these organizations have political links, which are more or less evident: in India, trade unions are traditionally associated with a particular political party; in the case of an NGO I talked to, the affiliation with the leading political party of the state was not mentioned, but became clear through party’s ideational support of the NGO of which I learned through digital media during online background research on the organization.

Part of the respondents initially were professionally involved in causes that were not directly related to emigrants’ issues but more generally about workers’ rights – similar to the Philippines (see Rother, 2022) – such as the domestic workers’ movement or advocating for issues of internal migrants from Northern India, who continue to be target audiences for these organizations. While working with the communities, for instance as social workers, they stated that they became aware of concerns of emigrants to the GCC countries, e.g. because domestic workers often would first work in India and then continue working abroad. In the case of Uttar Pradesh, the state’s demographic change towards becoming India’s primary emigrant state, led CSOs to start advocating for international emigrants. In several cases, the respondents had migrated to the GCC countries themselves, working in middle-class professions, and noticing the lack of state support upon return to India, decided to cater to the issues of international Indian migrant workers.

CSOs aim to advance the human, labour, and citizenry rights of emigrants and their families by influencing all stages of the policy process, particularly agenda-setting, policymaking, and implementation. Their focus is mainly on the pre-and post-migration stages and involves all institutional levels as well as migrants and their families. A rights-based framework entails foremostly ensuring safe migration practices (National Workers Welfare Trust, 2016). A key demand is to ensure better knowledge of and access to legal and social protection through extending the outreach of information on available legal services and government schemes, including their eligibility criteria and how to avail them, and by linking migrants with state and non-state service providers. Furthermore, in terms of implementing legal protection frameworks, claims include strict actions against recruitment agencies in cases of non-compliance with regulations; and strengthening and streamlining the capacities of Indian missions to respond to the needs of the migrants in distress. They demand equitable access to decent work opportunities by providing skill training and certification of their skills to prospective migrants; rehabilitation and resettlement opportunities to returnees; and government-to-government agreements, including internationally agreed standard contracts. For establishing robust social safety nets, CSOs ask, for example, the inclusion of international migrant workers in the 2008 Unorganised Workers Social Security Act and its welfare programmes; and measures to address missing migrants and deaths. In terms of political rights and representation, CSOs ask for migrants’ concerns to be taken up by political representatives of areas with high emigration rates to make it a salient political issue and voting rights for migrants abroad. Finally, CSOs appeal for the existing Emigration Act 1983 to be revised to include some of the above-mentioned measures.

## Invented spaces

This section discusses ‘invented spaces’ – independent platforms created by CSOs when excluded from official channels. It first highlights the role of transnational networks as a form of invented space in supporting Indian CSOs; it then highlights the gap between the Indian state and low-wage emigrants and their families, which CSOs leverage to claim a crucial role in service provision and policy influence. Third, it discusses how CSOs create invented spaces, including through social media and symbolic protest, for advocacy on the subnational level. Finally, this section describes how CSOs collaborate with local stakeholders like village councils (panchayats) to reach migrants and their families.

### Transnational alliances and networks

Many CSOs are part of transnational and national migrant and worker rights networks, such as union federations, but most prominently of the Migrant Forum in Asia (MFA). MFA is a regional network comprising migrants’ rights organizations and civil society actors in South Asia, Southeast Asia, East Asia, and the GCC countries. Piper and Rother (2022) have described the central role of MFA in facilitating the transfer of political advocacy, organizing strategies, and framing political issues promoting a rights-based approach to migration governance in Asia. The MFA secretariat coordinates network activities and facilitates horizontal learning among members and the multidirectional exchange of political ideas and practices, which Piper and Rother (2020) coin ‘political remittances’, across the transnational political space established by the network.

MFA is following a complementary ‘inside-outside strategy’ where it takes part and sends representatives to official migration governance events, such as meetings of the Abu Dhabi Dialogue (Piper & Rother, 2022) and the Global Forum on Migration and Development (Rother, 2009) and simultaneously creates events and demonstrations for civil society and migrants outside these venues to make their perspectives and voices more clearly heard (ibid.). Similarly, as the following sections will show, Indian civil society stakeholders use contestation, lobbying, participation, and cooperation strategies to reach stakeholders, such as local and state governments, the federal government, and IOs. Given that members of the MFA attend training that MFA provides, such as annual regional consultations (Piper & Rother, 2020), it can be assumed that these strategies also result from indirect or direct learning within the network. The majority of the respondents had either participated in trainings conducted by MFA and/or CSOs are partnering with the organization.

Transnational advocacy networks, such as MFA, are not only important sites of learning but also provide civil society stakeholders with higher visibility, access to wider publics and various governing sites (Keck and Sikkink 1998), such as international venues. For example, a representative of a South India-based CSO used their representation in the Global Forum for Development and Migration, the Colombo Process, and Abu Dhabi Dialogue on behalf of MFA to voice concerns to present representatives of the Indian state.^2^ In this way, invented spaces also function as a door to access the above-mentioned invited spaces of global migration governance. Civil society actors are aware of these network effects, particularly the impact of MFA: as a respondent from a CSO in Uttar Pradesh told me in a conversation about the future direction of their work, they would like to become an MFA member (Unrecorded conversation, 18 February 2023).

### Creating spaces of engagement in the absence of the state

A re-occurring topic in the interviews was a gap between service-providing institutions of the Indian state (see also Krishna, 2011) and international low-wage emigrants. Respondents from all states under study describe the distance between the state and its emigrant citizens resulting from the geographical remoteness of institutional migration support from rural areas:

The government [of Telangana] has now one or two facilitations centers in the center of Hyderabad. Telangana Overseas Manpower Company. But it is located in the headquarters. How many women from the villages will come there? They will not come. (Interview, 13 March 2020, Hyderabad)

State officials’ disinterest in engaging with migrants and their families is another reason causing a disconnect between state institutions and emigrants, as a respondent from a church-based organization remarks:

People don’t want to go to these offices [here NORKA ROOTS]. Immediately it may not be done. [The officers say]: ‘You can come another day’. Some officials are very helpful; some don't bother. They are concerned about their salary and working conditions; they are not at all concerned about people’s struggles. They [migrants] may be coming from other districts. (Interview, 22 February 2020, Trivandrum)

CSOs use the distance between state and citizens as entry points to claim that they occupy a crucial position in the governance of migration regarding service provisions:

In this, the role of the trade unions and civil society is equally important because the government’s reach is limited (…). The idea is that we work with different stakeholders to broad-base the campaign and to reach out to a large number of migrant workers in different areas of the country. (Interview, 3 February 2023, Online)

The absence of the state is decisive in the ability of CSOs to shape spaces of engagement, in particular invented spaces. In this study’s case of the CSOs, programmes, and services were often first established by the organizations and then duplicated by state institutions, often through collaborations, for example, on pre-departure training and information brochures. A respondent of a CSO in the South Indian state Kerala describes how they initiated a cooperation with NORKA, the Department of Non-Resident Keralite Affairs of the government of Kerala, which helped NORKA to reach its target audience for its programmes:

Initially, for example, the pre-departure orientation program of NORKA, they were conducting programs in different hotels, and they announced it in the newspaper or maybe on their website. But who has access to this? And then a hundred or two hundred Rupees registration fee. Our people asked, ‘Why should I pay 200 Rupees and go like that’? They will not; very few participated – only the educated people. But the unskilled labor force never goes for this kind of thing. There only our intervention was making a difference. We organized many [pre-departure] programs at the [Keralan] coastal belt. And NORKA would come there [with us] and give awareness. We act like a bridge between the people and the government. (Interview, 22 February 2020, Trivandrum)

I argue that providing services to migrants, such as pre-departure training and information about the migration process, are vehicles of norm promotion, where CSOs frame agendas and policies concerning migrants through their programmes. This means that CSOs do not simply implement policies but shape and diffuse policies from the bottom up. These findings highlight the proactive and participatory role of CSOs in the policy process, emphasizing their capacity to influence and promote policies. As one respondent in Kerala recalls: ‘I did a lot of pre-orientation training for the trainers for both of the governments [Andhra Pradesh and Telangana]. After that, they actually got involved with the workers’. (Interview, 13 March 2020, Hyderabad).

The Indian federal state institutionalized the pre-departure trainings in 2018, while CSOs in Kerala and Telangana already conducted them in 2003 and 2016.^3^ Establishing their presence through services also explains the continuing programmatic focus of many CSOs on pre-departure training, which might not be the most obvious rights enforcement scheme as they are often viewed as a measure of migration facilitation rather than protection (see, e.g. Chee, 2020). Furthermore, based on the information brochures provided to potential migrants during the training, CSOs are communicating the rights of migrants and how to access them much more explicitly, for example, by stating ‘[y]ou have a right to reject extra working hours in a day’ National Workers Welfare Trust 2016) and ‘[y]ou have a right to be free from torture, humiliation and oppression’ (ibid.), and explaining how to access insurance coverage. On the other hand, the edition of the federal government limits its awareness raising regarding rights to stating to domestic workers that they have the right to an environment free from sexual harassment and violence and listing whom to contact when in trouble (India Centre for Migration and UN Women, 2018).

### Inventing new spaces on the subnational scale through demonstrative participation

CSOs create new, invented spaces of demonstrative political participation using technologies and social media and symbolical forms of protests (see Kersting, 2013). In line with Bisong’s (2022) findings, these invented spaces are created to circumvent restrictive spaces of political representation and participation:

One person died in Dubai due to ill health, and the coffin reached Hyderabad airport. From Hyderabad airport to his village, while traveling in an ambulance, our Gulf activist stopped the ambulance on the way out of [location]. They put the coffin before the MLA’s [Member of Legislative Assembly] residence gate: They raised their voices for 15 min, conducted a Facebook Livestream, and discussed. (…). It recently happened three days ago. Why? Because we failed all the other ways. The government is not listening. This is the only way to attract the media, the public, and the government. (Interview, 17 February 2023, Online)

The described unconventional form of protest stands in connection with migrant activists’ demand in the South Indian State of Telangana to expand the current federal government-run compulsory insurance scheme called *Pravasi Bharatiya Bima Yojana* (2003) to cover death due to natural causes and not only accidental death and permanent disability. The argument here is that often deaths, which occur during working hours and possibly due to unsafe working conditions, are claimed as natural deaths.^4^ Consequently, the deceased’s families do not receive a cover sum, nor are costs for repatriating the dead body covered by the insurance. Representatives of CSOs located in Telangana/Hyderabad claim that even though the current party in power in the state of Telangana promised to cater to migrants’ issues, such as providing a budget for emigrants’ welfare and the families of deceased migrants, in their election manifesto in 2014, the same election which also decided the bifurcation of the state Andhra Pradesh to create Telangana, these promises have not transformed into tangible action.^5^

As the protest in Telangana shows, the mobilization of public opinion and awareness creation through public events is more likely to take place on the subnational scale as migration as a public policy issue has higher political traction for regional governments than for the Indian federal state (see Burmeister-Rudolph, 2023). Similarly, as the next sections demonstrate, CSOs make use of the remoteness of the state in rural areas (Krishna, 2011) and decentralized political structures to create invented spaces that fill ‘governance gaps left by the state’ (Bisong, 2022, p. 2955), such as working together with local rural CSOs to raise awareness about the migration process. They also refer to the typical repertoire of inside strategies, such as providing direct engagement with decision-makers through meetings to discuss their interests and concerns, policy expertise to help inform decision-making, and conveying the views and needs of the groups they represent. Importantly, the resulting associations with state actors are partnerships. CSOs influence the policy process by agenda-setting activities and practices, which are adopted by state institutions.

### Scaling down: collaborations at the grassroots

A common inside strategy for CSOs in all four states has been to seek collaborations with relevant stakeholders at the lower administrative levels, such as village- or district levels:^6^ The CSOs are located in urban areas and depend on these cooperations to reach migrant populations. For example, an essential part of a visit with a CSO to a rural district approximately two hours away from Lucknow, Uttar Pradesh’s capital, which I accompanied, was to meet with a local NGO and with the head of the elected town/village council, the panchayat. The panchayat members knew where families of migrants or returned migrants lived and introduced us to them. Representatives of the CSOs asked them about their concerns and told them about the national support programmes, which migrant and their families were not aware of. The visit ended with us meeting the Pradhan, the village head. It was clear this was mandatory, not only because of the panchayat’s knowledge about who in the village were migrants but also to establish a good relationship with the village council members. During the visit, a CSO representative explained that reaching out to the panchayat was their usual procedure. CSOs in other districts and states also use the ‘scaling down’ strategy to disseminate their agenda. A respondent in a central migrant origin district in Uttar Pradesh recalls a similar approach:

[T]hese people move out from rural areas. (…). We are on a move to sensitize the (…) Pradhan, who is the head of that panchayat, so that he is aware of the people who are moving out (…) either interstate or internationally, for work purposes or any other purpose. He should also be aware of it so that the people are safe. And he has a record of how many people there are. So we also sensitize them on these issues. (Interview, 7 March 2023, Online)

A respondent of a migrant support organization in Kerala narrates how the involvement of local governments in raising awareness about the migration process is relevant. Moreover, given the high prevalence of migration from Kerala to the GCC over the last five decades, many panchayats are former migrants themselves, making it easier for CSOs to cooperate with them:

Now we have this three-tier system, panchayat, village level, block level, as well as district or state level. The panchayat cooperations are more aware of it. We found that many of the councils at the panchayat level are returnees. We met many panchayat board members at the village or municipality levels who are counselors. Many of them have worked many years in the Gulf, so they know the situation. That is also helping us to get to the people. They will know issues faced by the unskilled labor, the labor camps, and the situation [there]. Some of them have experienced this themselves; at least they have seen [it]. So when we ask them, ‘Shall we organize an awareness program, an orientation for those’, we get their support also. That is one positive experience we have. (Interview, 22 February 2020, Trivandrum)

The same organization also anchors its work, which is more focused on aspects of economic livelihoods and financial stability, in other decentralized governance structures specific to the state of Kerala, such as the Keralan state’s poverty eradication programme *Kumdumbashree*:

Initially, we formed the groups, and they used to meet once in a month. We promote their [migrant families’] savings. Otherwise, whatever they earn abroad, send to the family, it is lavishly spent here, and finally, when they come without any job or lose their job, they have to start from zero again. (ibid.)

The programme, initiated in 1998, works through women’s neighbourhood groups and is based on the participatory governance structures initiated by the ‘People’s Campaign for Decentralized Planning’ (Williams et al., 2011). In 1993, the Indian central government amended the constitution to devolve budget authority and mandate direct participatory structures and processes to local governments, sparking the campaign. Over 100,000 ‘key resource persons’ were trained at the local and district levels as part of the institutional reforms, with an emphasis on women and persons of Scheduled Castes/Scheduled Tribes (Heller et al., 2007).

A migrant rights organization located in Hyderabad, the state capital of Andhra Pradesh and Telangana, views working through already established decentralized institutions as similarly beneficial but claims that the governments would not take such initiative. Instead, it was the organization that made use of its local networks to conduct pre-departure training for female domestic workers who were planning to migrate:

They [governments of Andhra Pradesh and Telangana] could also work with other networks because the government has its system of working at the grassroots. There are self-help groups or ASHA [accredited social health activist] workers. These networks should be made use of to reach the grassroots workers. Again, this is not a priority; they [the state government] did not reach out to many people in Andhra. But in Telangana, we took the responsibility because the government was not reaching out (…). I went and challenged them. I said, ‘You have to do this’. Because I knew that this project [financing through UN Woman] had come, and they were not doing anything. We also went around and called other organizations. Together we reached out to more than 3000–4000 women through our network. (…) Besides, we are also looking at returnee migrant associations, creating returnee migrant associations, where they would become partners in creating awareness. (Interview, 13 March 2020, Hyderabad)

## Instrumentalized spaces

This section discusses ‘instrumentalized spaces’ – collaborations on migration issues between CSOs and IOs in India to utilize each other to achieve their own goals. Nevertheless, collaborations are often viewed mutual beneficial from both stakeholders: CSOs need resources and IOs need implementation. They allow IOs to spread their migration frameworks (top-down) and CSOs to use successful programmes to pressure governments for more involvement (bottom-up).

### Seeking collaborations with international organizations

CSOs turn to IOs to finance their projects and for infrastructural support, as the previous excerpt exemplifies. Due to low responsiveness, non-existing service provisions and institutions for the management of migration by the majority of the Indian states and the federal state, CSO representatives describe how they turn to collaborations with IOs, such as the ILO, UN Woman, IOM, and MFA. These collaborations not only enable CSOs to conduct their programmes but also pave the way for IOs to implement their migration frameworks in the daily work of organizations that are active on the ground. Similarly, to CSOs, bypassing governments can mean for IOs a quicker turnaround time for the implementation of programmes. As a result, these collaborations act as sites of norm diffusion top-down through the IOs and bottom-up, as CSOs can use their successful programmes as a platform to encourage governments towards higher levels of involvement and the presence of IOs to create visibility and urgency of the topic to push for policy change. Accordingly, CSOs are not only instrumentalized, but they utilize these spaces for their purposes (see also Marino et al., 2023). A representative of a CSO located in the South Indian city of Hyderabad recalls:

We went to UN Women. UN Women asked me to prepare some 30 sessions on safe migration. We prepared those sessions. Then the government came in. With UN Women and the ILO together, we proposed preparing a state migration policy. That is for Andhra separate, Telangana separate. Finally, the government of Andhra has a policy on migration, whereas the Telangana government has prepared and announced it, but it is still not a priority to them. However, after our work, UN Women began to work with these two governments on the migration issue, and they also gave many funds to the government to organize. I did a lot of pre-orientation training for the trainers for both governments. After that, they got involved with the workers. (Interview, 13 March 2020, Hyderabad)

Another example is the establishment of a migrant resource center in the city of Gorakhpur, in Eastern Uttar Pradesh, a region with high numbers of emigrants to the GCC countries, which the IOM supports by funding staff of the centre. The IOM initiated the cooperation, illustrating how invited spaces serve the purpose of propagating certain policies, and thus become instrumentalized, as described by Bisong (2022). The support of the IOM goes beyond funding but also involves disseminating content:

The Migrant Resource Centre, which we are running, is in association with the International Organization of Migration. The IOM has supported us in establishing this. So, we make a common thing. We mutually designed [the information brochures] and got them approved by IOM. We design it, and then we sometimes take permission from the government and the state government to use their logos. And secondly, in terms of pre-departure training, the module we have developed is in collaboration with IOM India and [name of the CSO]. (Interview, 7 March 2023, Online)

This example underscores the role of civil society as catalysts and facilitators in promoting particular ‘philosophies of migration’ (Pécoud, 2021). As with the collaboration between the IOM and the Uttar Pradesh-based CSO, this suggests a managerial or developmental approach influences the organization’s practical work. The IOM has been in particular critized of promoting this approach and the related objectives of safe, orderly, and regular migration, which is often described as being in opposition to rights-based migration governance (Pécoud, 2021; Piper & Rother, 2012). Indeed, one of the defining keywords ‘safe migration’ features prominently in the description of the Migrant Resource Center:

[Name of the CSO] & IOM have jointly agreed to work upon the theme of promoting *safe* migration and creating *safe* working environment for migrant workers (…). Efforts are being made to benefit the migrants from the Migrant Resource Center so that along with promoting *safe migration*, migrants can be made aware and *safe* from the heinous crime like human trafficking and forced labour. (Manav Seva Santsthan, 2022, emphasis added)

Critics argue that the managerial/developmental approach can downplay the importance of migrants’ rights by focusing primarily on safety and operational efficiency (Pécoud, 2021). This terminology acknowledges the well-being of migrants but arguably in a superficial and non-binding manner (ibid.). Compared to West African countries (see Marino et al., 2023) or the Philippines (Gardiner Barber & Bryan, 2017), for example, IOM’s involvement in India’s migration governance is (still) limited. India has been a member state of the IOM since 2008, thus its membership is comparatively recent (IOM UN Migration 2024). It has therefore yet to be seen how the organization’s growing presence will play out in terms of its potential influence on CSOs in adopting managerial/development agendas.

## Invited spaces

This section focuses on how CSOs navigate formal platforms and institutions created by the Indian state, ‘invited spaces’ of engagement. Their key strategies include lobbying legislators; using existing advocacy networks on related issues; engaging with the Protector of Emigrants; advocating for legal reform; and supporting political participation of migrants and their families.

### Between advocacy and collaboration with regional and national legislators

As a standard for inside strategies, Indian civil society actors aim to target members of parliament – particularly from areas (districts) with a high percentage of emigrants^7^ – to promote a rights-based migration framework.^8^ The Indian political administration is federally organized, including a national parliament and regional parliaments, the Legislative Assemblies. Among a few other states, Andhra Pradesh, Telangana, and Uttar Pradesh also have a State Legislative Council. Approaches include the distribution of informational leaflets^9^ and organizing official meetings with Members of the Legislative Assembly (regional) and Members of Parliament (federal).^10^

Organizations that support issues concerning GCC-bound migrants draw on the strategies of issue-linkage related to labour rights and transnational network membership to scale up their outreach, that is, to get in contact with and convey their views to policy and decision makers on national and international scales. As described earlier, several CSOs moved from working more generally on workers’ rights and topics of social inclusion to incorporating emigrant workers as they saw an overlap of shared issues. Already having a platform through previous social movements and advocating efforts makes it easier to establish additional claims for Indian domestic workers who work abroad:

We regularly campaigned, lobbied, and advocated for the domestic workers’ rights for a national policy, a comprehensive legislation. We worked on the state laws here in our state and brought minimum wages for the workers. So that helped us. We said it is not only the domestic workers [in India], but the government must also address the workers abroad as domestic workers. We asked to include them in the national policy. They [the Indian government] might not have control over [what happens abroad], but they are domestic workers. They should also be included. (Interview, 13 March 2020, Hyderabad)

On the subnational scale, relevant civil society stakeholders address the Protector of Emigrants (PoEs) offices, administratively falling under the Indian MEA. The PoEs oversee and issue migration clearances for low-wage workers migrating to the GCC countries, including checking for proper working contracts and employment regulations; and registering recruitment agencies. Thus, they are central stakeholders in checking for commitment to labour rights and fair recruitment processes, at least on paper. Regional offices of the PoE are represented, among other states, in Kerala, Uttar Pradesh, and Hyderabad (for Andhra Pradesh/Telangana).

Foresting good relations with the PoE is a means for CSOs to reach migrant workers who migrate via the official route and recruitment agents as either migrants themselves and/or the respective recruitment agent come to the PoE to obtain the emigration clearance. Moreover, for migrant workers, especially female migrants, who often migrate for domestic work, CSO actors aim to create awareness about the migration process during the waiting time at the office, which they claim recruitment agencies would not provide.^11^ For the same reasons, the CSO approached the state’s Women’s Commission.^12^ An activist located in Hyderabad recalls the encounters with recruitment agents and with the PoE in Hyderabad:

We went and met the PoE. In the beginning, he was not willing [to meet us]. Over time, we have developed much material on safe, orderly, regular migration and how women must be prepared. So later on, we entered into an MOU with the government and the Protector of Emigrants, and he allowed me to come twice a week [to the POE’s office] and speak to the women. So earlier, they were not allowed to sit inside. But after our MOU, the women were allowed to sit inside, but the recruitment agents were not allowed inside. So that was good; we could speak freely to the workers. (Interview, 13 March 2020, Hyderabad)

The interventions and the absence of the recruitment agents and their families allowed some women to articulate that they were migrating against their wishes.^13^ Simultaneously, being connected with the PoE allowed the CSO to reach out to recruitment agencies and discuss with them fair recruitment practices.^14^

A point of contention and criticism of many CSOs who support emigrants’ rights has been the 1983 Indian Emigration Act 1983, through which the Indian state regulates emigration. There have been several attempts to overhaul the law, which also has been a demand by CSOs:^15^ ‘We have the [Emigration] Act of 1983. We say the central government must create something new and amend it. So far, it is not enforced. The act is very outdated’. It has been criticized for not taking into account temporary developments regarding migration to the GCC countries, e.g. increasing numbers of female migrants in the last two decades, but also for creating a discriminatory practice as individuals with a high school education below class 10 who plan to migrate to countries in the GCC region, as well as several other countries in Southeast Asia and the Middle East, need a so-called emigration clearance without which they are not allowed to leave India.

Some CSOs under study are proposing to amend the bill to include a rights-based framework that protects emigrants’ rights through their enshrinement in law. According to a CSO located in Kerala:

We need proper laws, so we can challenge them and approach the court. Unless that is there, whatever we do is not perfect. We found this is the gap. We need to discuss these gaps. (…). So, we need to stand together to pressure the government to advocate for it [amendment of the Emigration Bill]. The rights we have to lobby for it. (…) We try to press the government to intervene in issues. Also, to adopt proper legal measures. We meet the MPs, and we convince them, we educate them or try to make it clear to them because (…) this does not become a focus unless someone is behind that. (…). It is also awareness campaigns, where we call them, the MPs or ministers or MLAs so that they become aware of the difficulties people face. (Interview, 22 February 2020, Trivandrum)

In 2021, the MEA proposed replacing the 1983 Emigration Act with the 2021 Emigration Draft Bill (MEA 2021). The MEA invited feedback on the draft from the public, including CSOs, before relegating it to the Indian parliament. A representative of a union federation, which represents 49 Indian trade unions, recalls their involvement in providing suggestions and observations:

[We provide feedback] [u]sually not just for the emigration bill, but for any act of [labor] policy that is coming up. (…). The government comes up with a draft, and they seek or invite suggestions and feedback (…). So, as a global federation, we organize consultations with the trade unions. This emigration [bill] was during COVID time, so we had an online consultation (…). Then we try to submit points for each [section] line by line or paragraph by paragraph. (Interview, 3 February, Online)

The union federation advocates for a framework that strengthens human, political, and labour rights. One of the demands is a clear definition of abuse, exploitation, or violation of migrant workers’ rights within the Emigration Act. Furthermore, the union federation asks for voting rights for migrant workers, that is, the possibility of postal voting or voting in Indian embassies abroad. Furthermore, it suggests including a model labour contract in the bill’s ambit. Importantly, it proposes to strengthen the self-representation of migrant workers in newly proposed policymaking and implementing bodies, such as the Bureau of Emigration and Planning, Nodal Committees of States and Union Territories, and Emigrant Welfare Committees associated with Indian embassies. Also, the union federation is in favour of putting safeguards in place for undocumented migrants, in particular for those who overstayed their work visas (Unpublished document, Indian Affiliates Council, 3 June 2021).

The listed demands exceed the call for human and labour rights and aim to include direct (voting) and indirect (self-representation) political rights. They imply a redefinition of the citizen-state linkage, where (e)migrants are involved in rights-producing politics. Similarly, rooting social rights in law, e.g. by establishing Indian embassies’ welfare wings and defining their responsibilities instead of leaving social assistance distribution to the discretion of social protection programmes, would mean a transformation of beneficiaries or clients into citizens who can claim their rights: ‘in a policy framework, citizens are mere beneﬁciaries, but rights are entitlements that citizens can demand’ (Jha, 2019, p. 86).

Although, at the point of writing, the future of the 2021 Emigration Bill and its final content is unknown – neither the MEA provides information about its current status nor is it listed under the bills list of the Ministry of Parliamentary Affairs, some of the earlier demands of the union federation concerning the registration of local sub-agents of recruitment agencies had been incorporated:

In 2010, one of our key demands was to include sub-agents. In this current draft, they mention sub-agents, although their roles and responsibilities have not been outlined. That was one of our recommendations when we submitted our presentation [in 2021]. So we cannot say that it [the final bill] is entirely different or completely the same until there has been movement [presenting the bill in parliament]. (…). So from 2010, definitely there have been inclusions of certain good points in the current draft. (Interview, 3 February 2023, Online)

Lastly, CSOs have supported migrant families to participate in spaces of representative democracy by encouraging their candidature in elections. While research has shown that returnees and migrants exert significant influence within Kerala’s major political parties through affiliated organizations and serve as influential lobbying bodies (Akhil & Ganga, 2022), not much is known about the effects of migration on political processes in other Indian states. There are indications, however, that increasingly emigrants and their families take influence and actively participate in the electoral process beyond Kerala. For example, in Telangana, for the upcoming state election in 2023, family members of Gulf migrants, with the support of CSOs, aim to contest in the election to signal dominant parties to take their previous electoral claim to support emigrants’ welfare seriously (Rao, 2023).

## Conclusion

This article examined the spaces for engagement and the strategies of Indian civil society stakeholders located in four major Indian emigrant-origin states in the advancement of emigrants’ rights. It showed they vary between inside advocacy and outside activist strategies when seeking to influence policymaking and adjust these strategies to utilize the opportunity structures a multi-level, multi-stakeholder political space offers. CSOs’ engagement and strategies are mediated by subnational variations in the presence of the migration state.

Importantly, contributing to the literature on local migration governance by bringing in empirical perspectives on emigration states, this research demonstrated the centrality of local scales and collaborative efforts to work together with local political stakeholders in their promotion of migrants’ rights due to easier access to public stakeholders and the state’s predominant absence in rural, peripheral regions (see Krishna, 2011). Through scaling their activities to local levels, CSOs create invented spaces for engagement. Lobbying and awareness campaigns with village and municipal councils and providing service to migrants, such as pre-departure training, allows civil society actors to act as policy entrepreneurs and establish particular agendas and practices that are anchored in rights-based approaches to migration. Indian CSOs also do ‘scale jumping’ (MacKinnon, 2011, p. 24) to make use of invented spaces which transnational migrant rights networks provide to increase their outreach and gain allies.

Strategies targeting IOs are geared towards cooperation and lobbying as CSOs depend on their funding, networks, and IO’s possible impact on government stakeholders. IOs also utilize these cooperations to place their agendas in the workings of the CSOs, particularly those who are involved in service provision for (potential) emigrants and their families. This research highlights how local CSOs become arenas where often conflicting norms of how to govern migration converge as they are often part of transnational migrant rights networks yet cooperate with IOs, such as the IOM and UN Woman. These findings showcase the fluidity between invited and instrumentalized spaces, where advocacy and depoliticizing co-optation go together (see Ålund & Schierup, 2018).

Civil society actors utilize invited spaces where they deploy inside strategies, such as direct lobbying and policy feedback, as well as they create invented spaces through outside strategies, such as public demonstrations and the mobilization of public support to target regional stakeholders, such as members of the Legislative Assemblies or the regional PoEs, as these are the institutions that are concerned with the day-to-day implementation of national policies and policymaking for the respective states. Political stakeholders at the national level become relevant for federal legislation, e.g. reforming the Emigration Act or the Domestic Workers Act, where CSOs use invited spaces to influence legislative reforms. The data suggests that the choice of strategies also depends on political opportunity structures on each scale and the responsiveness of political stakeholders, which again highlights the importance of considering regional and local perspectives when studying a federally organized state, such as India. Similar to other geographical contexts and scales (see Bisong, 2022; Soykan & Şenses, 2018), invited spaces are ultimately selective in who is invited, thereby including certain voices while excluding others. Often the excluded voices are migrant voices themselves which points to the more general problematic of CSO’s legitimacy in representing (e)migrants’ rights.

Finally, this article shows that – differently from what scholarship has described – definitions of social justice in the Indian context exceed human and labour rights, but also emphasize representational and citizenship rights, including political representation, broader social benefits, and greater accessibility to public services. These insights call for further research on how specific national and local political contexts and constellations in other regions shape the dynamics and demands of civil society stakeholders in their strife to advance emigrants’ rights.

## Notes

1 CSOs include ‘all non-market and nonstate organizations outside of the family in which people organize themselves to pursue shared interests in the public domain’ (Tomlinson, 2013).

2 Interview, 13 March 2020, Hyderabad.

3 Interview, 22 February 2020, Trivandrum.

4 Interview, 17 February 2023, Online.

5 Interview, 17 February 2023, Online; Interview, 13 March 2020, Hyderabad.

6 Interview, 7 March 2023, Online.

7 Interview, 13 March 2020, Hyderabad.

8 Interview, 7 March 2023, Online; Interview, 17 February 2023, Online; Interview, 13 March 2020, Hyderabad.

9 Interview, 13 March 2020, Hyderabad.

10 Interview, 13 March 2020, Hyderabad.

11 Interview, 13 March 2020, Hyderabad.

12 Interview, 13 March 2020, Hyderabad.

13 Interview, 13 March 2020, Hyderabad.

14 Interview, 13 March 2020, Hyderabad.

15 Interview, 3 February 2023, Online.
